# Deficiency of IL-7R attenuates abdominal aortic aneurysms in mice by inhibiting macrophage polarization towards M1 phenotype through the NF-κB pathway

**DOI:** 10.1186/s10020-025-01209-2

**Published:** 2025-04-16

**Authors:** Shengnan Xu, Xueyu Han, Yi Yu, Chuan Qu, Bo Yang, Bo Shen, Xin Liu

**Affiliations:** 1 Department of Cardiology, Renmin Hospital of Wuhan University, Wuhan, 430060 P.R. China; 2 Cardiovascular Research Institute, Wuhan University, Wuhan, 430060 P.R. China; 3 Hubei Key Laboratory of Cardiology, Wuhan, 430060 P.R. China

**Keywords:** Abdominal aortic aneurysm, Macrophages, M1 macrophage polarization, IL-7/IL-7R, NF-κB pathway

## Abstract

**Background:**

Abdominal aortic aneurysm (AAA) is a common degenerative disease of the abdominal aorta, which can result in extremely high mortality owing to the rupture of the abdominal aorta. The activation of IL-7R has been shown to modulate the inflammatory responses, which play an important role in the progression of AAAs. However, the mechanism of IL-7/IL-7R axis in AAAs is still unclear.

**Aims:**

This study aims to investigate the effects of IL-7R on AAAs and the underlying mechanisms involved.

**Methods:**

Wild-type C57BL/6 and IL-7R knockout mice were used as experimental subjects. ELISA analysis, histological staining, western blotting and qPCR were performed to explore effects of IL-7R deficiency in the formation and development of elastase-induced AAAs. Transwell, CCK8, and immunofluorescence assays detected the migration and polarization of RAW264.7 macrophages in vitro.

**Result:**

We demonstrated that IL-7R was elevated in mice with AAAs. Blocking IL-7R can inhibit the formation of AAAs and reduce aortic dilatation, elastic layer degradation, and inflammatory cell infiltration. Knockout of IL-7R suppressed the migration, infiltration and M1 polarization of macrophages. Moreover, inhibition of the NF-κB signaling pathway by BAY 11-7082 attenuated the macrophage-mediated inflammatory responses caused by IL-7R overexpression.

**Conclusion:**

In short, this study showed that IL-7R promotes the infiltration and migration of macrophages by regulating M1 macrophage polarization, possibly in part via activation of the NF-κB pathway, which may be associated with the development of AAAs.

**Graphical abstract:**

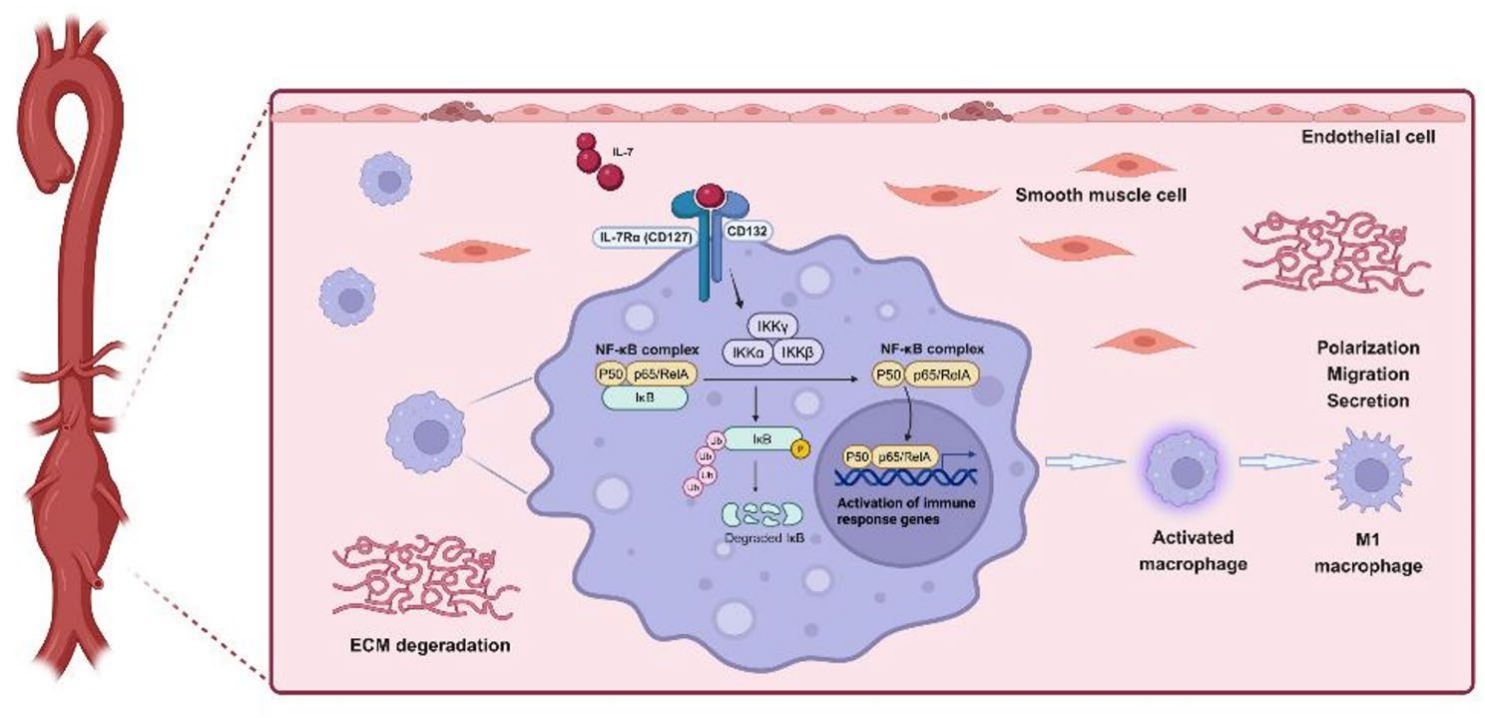

**Supplementary Information:**

The online version contains supplementary material available at 10.1186/s10020-025-01209-2.

## Introduction

Abdominal aortic aneurysm (AAA), occurring primarily in men aged ≥ 65 years, is a common degenerative disease of the abdominal aorta that can lead to dilatation and rupture (Sakalihasan et al. 2005; Baman and Eskandari 2022; Golledge et al. 2023). Most AAAs are asymptomatic until rupture, which are usually life-threatening and have a mortality rate of 65–85% (Hellenthal et al. 2009, 2018). In case of larger AAAs, typically ≥ 5.4 cm in diameter, or symptomatic AAAs of any size, open surgery or endovascular aortic repair is effective in preventing death caused by the rupture of the aneurysms (Schanzer and Oderich et al. 2021; Golledge et al. 2019). However, owing to the limited understanding of the mechanisms underlying the initiation and development of AAAs, treatments for small and asymptomatic AAAs are lacking (Golledge et al. 2019; Molacek et al. 2020). It is important to explore the pathophysiologic mechanisms of AAAs and identify relevant therapeutic targets.

The pathological features of AAAs include chronic aortic inflammation (Raffort et al. 2017), vascular smooth muscle cell (VSMC) apoptosis (López-Candales et al. 1997), extracellular matrix (ECM) degradation (Cohen et al. 1992), and increased matrix metalloproteinase (MMP) levels (Salarian et al. 2023). Over the past decade, studies on clinical samples and animal models have shown that the infiltration and polarization of macrophages play a critical role in the occurrence and progression of AAAs (Raffort et al. 2017; Salarian et al. 2023; Song et al. 2022; Davis et al. 2021; Huanggu, Yang 2023). Monocytes in peripheral blood are recruited to the aortic lesion site and then differentiated into macrophages, which are the primary source of macrophages in an aortic aneurysm (Yuan et al. 2020). Macrophages are classified into two subsets as follows: classically activated (M1) macrophages and alternatively activated (M2) macrophages (Song et al. 2022). M1 macrophages can produce high levels of proinflammatory cytokines (e.g., interleukin [IL]-1β and inducible nitric oxide synthase [iNOS]) and proteolytic enzymes, leading to aggravation of local inflammation, loss of VSMCs, degradation of ECM, and pathological dilatation of the aorta (Batra et al. 2018; Zhang et al. 2024; Shi et al. 2020). On the contrary, M2 macrophages exert anti-inflammatory effects and are associated with ECM remodeling and tissue repair (Raffort et al. 2019; Pope et al. 2016).

Studies on IL-7, a pro-inflammatory cytokine, have mainly focused on its role in T and B cell lymphopoiesis, thymocyte maturation, and mature T cell homeostasis (Desvaux et al. 2023). Upon its binding to IL-7, the IL-7 receptor (IL-7R) can activate various intracellular signaling pathways, such as the nuclear factor kappa-B (NF-κB) pathway (Qu et al. 2016). The IL-7/IL-7R pathway exerts an important influence on regulating the inflammatory response (Meyer et al. 2022; Kim et al. 2020; Xu et al. 2021; Willis et al. 2012). Recent studies have shown that macrophages are the main effector cells in IL-7-induced arthritis (Kim et al. 2020). Activation of IL-7/IL-7R enhances the cytotoxic activity of macrophages and promotes lots of pro-inflammatory cytokines secretion (e.g., monocyte chemoattractant protein [MCP]-1, tumor necrosis factor-alpha [TNF-α], and IL-1β) by macrophages (Yan et al. 2021; Bao et al. 2018). However, the specific mechanisms through which the IL-7/IL-7R pathway affects macrophages and the roles of different macrophage subsets in aortic diseases remain unclear.

In this study, we investigated the effects of the IL-7/IL-7R signaling axis on macrophages and AAAs. IL-7R deficiency improved the development of AAA in mice and attenuated inflammatory macrophage infiltration in the lesion tissue. Our results showed that down-regulating IL-7R expression in macrophages inhibits the polarization of macrophages toward the M1 phenotype and attenuates their migratory capacity, which may be partly mediated by restraining the activity of the NF-κB pathway.

## Materials and methods

### Animal experiments

A total of 34 male C57BL/6 mice (8 weeks old) were purchased from the Animal Experimental Center of Three Gorges University (Wuhan, China). The IL-7R knockout (IL-7R KO) mice on the background of C57BL/6 and their wild-type (WT) littermates were obtained from Wuhan Youdu Biotechnology Co., Ltd. for the construction of the AAA model. All mice were housed in the animal management center of the Third Hospital of Wuhan City (No. SY2023-041) with a 12-hour light-dark cycle. Mice were kept under specific pathogen-free, environmentally controlled (temperature 20–25 °C; humidity 50%±5%) conditions, with cages individually ventilated and food and water supplied ad libitum. All animal experiments followed the *Guide for the Care and Use of Laboratory Animals* (NIH Publication No. 82 − 23, revised 1996).

In the first part of the animal experiment, C57BL/6 mice were randomly divided into three groups as follows: control group (*n* = 8), PPE (Elastase, from porcine pancreas) + IgG (a rat IgG2a isotype mAb, from BioXcell, New Hampshire, USA) group (*n* = 13), and PPE + anti-IL-7Rα (clone A7R34, from BioXcell, New Hampshire, USA) group (*n* = 13). In the second part, IL-7R KO mice and their WT littermates were randomly divided into the control (KO, *n* = 9; WT, *n* = 9) and PPE groups (KO, *n* = 21; WT, *n* = 21). The AAA model was established as described in previous studies (Zhai et al. 2022; Zhao et al. 2022; Bhamidipati et al. 2012). Briefly, mice were anesthetized via intraperitoneal injection of pentobarbital sodium (50 mg/kg, i.p., Sigma-Aldrich, St. Louis, USA) and subsequently subjected to open abdominal midline surgery to expose the infrarenal abdominal aorta. The infrarenal abdominal aorta was wrapped with a sterile filter paper pre-soaked in 2.5U PPE (E1250, Sigma-Aldrich, St. Louis, USA) for 30 min. After the filter paper was removed, the abdominal cavity was washed twice with sterile saline and sutured. In the control group, the infrarenal abdominal aorta was wrapped with a filter paper pre-soaked in PPE inactivated at 100 °C for 30 min, and the subsequent procedure was the same as that in the PPE group. In addition, mice in the PPE group were administered 0.1% β-aminopropionitrile (BAPN, A3134, Sigma-Aldrich, St. Louis, USA) in drinking water beginning from 2 days before surgery until 14 days after surgery. The mice in the PPE + IgG and PPE + anti-IL-7Rα groups administered IgG2a isotype mAb or anti-IL-7Rα mAb (20 µg/mouse/day) after surgery, respectively. After 14 days of exposure to PPE, the mice were anesthetized (pentobarbital sodium, 150 mg/kg, i.p.) and euthanized to collect their blood and organs.

### Plasma collection and histological analysis

After the mice were anesthetized with pentobarbital sodium, blood was collected from the inferior vena cava in fresh EDTA tubes. The blood samples were centrifuged at 3000 g for 15 min at 4 ℃ to obtain plasma. In addition, the abdominal aorta was carefully exposed and isolated under an Olympus microscope. The maximum diameter was recorded and quantified using Image J software. The AAA was defined as localized arterial dilation to more than 50% of the mean abdominal aortic diameter of the corresponding segment in the normal mice (Yang et al. 2023). The plasma and some aortas were stored at -80℃, whereas the remaining aortas were fixed with 4% paraformaldehyde for 24 h at room temperature, embedded in paraffin, and sectioned at a thickness of 5 *µ*m. After paraffin removal, elastin Van Gieson (EVG, G1042, Servicebio, Wuhan, China) staining was performed according to standard experimental procedures to assess degradation of the medial elastic layer. The elastin degradation was described as four grades based on the degree of elastin fibers break: grade 1 (less than 25% degradation), grade 2 (25–50% degradation), grade 3 (50–75% degradation), and grade 4 (over 75% degradation) (Yang et al. 2023; Zhao et al. 2017). Images were collected with a digital slide scanner (Pannoramic 250/MIDI, Hungary) and analyzed with the Image J software (NIH, Bethesda, MD, USA).

### Enzyme-linked immunosorbent assay

Enzyme-linked immunosorbent assay (ELISA) kits were used to evaluate the concentrations of IL-1β (PI301, Beyotime, Shanghai, China), c-reactive protein (CRP, PC186, Beyotime, Shanghai, China) and IL-7 (EK207, Multi Sciences, Hangzhou, China) in plasma according to the manufacturer’s instructions.

### Immunohistochemical staining

For immunohistochemical assay, abdominal aortic Sect. (5 *µ*m) were dewaxed in xylene, and CD68 expression was detected according to the manufacturer’s instructions. The tissue slides were incubated with primary antibody against CD68 (1:200, GB113109, Servicebio, Wuhan, China) overnight at 4°C. After washing excess unbound primary antibodies, the slides were incubated with HRP-conjugated goat anti-rabbit IgG (1:200, GB23303, Servicebio, Wuhan, China) and stained with 3,3’-diaminobenzidine (DAB). Finally, the sections were stained with hematoxylin (G1004, Servicebio, Wuhan, China) and observed under a light microscope. Images were recorded using a digital slide scanner (Pannoramic 250 FLASH, Budapest, Hungary), and the expressions of CD68 were quantified via the image J software.

### Immunofluorescence staining

For double immunofluorescence staining, dewaxed abdominal aortic sections were fixed with 100% cold methanol and non-specific binding sites were blocked with 1% bovine serum albumin. These sections were incubated initially with anti-IL-7R antibody (1:200, TD6362, Abmart, Shanghai, China) and Cy3-conjugated goat anti-rabbit IgG (1:500, GB21303, Servicebio, Wuhan, China), subsequently with primary antibodies against CD31 (1:500, GB120005, Servicebio, Wuhan, China), α-smooth muscle actin (α-SMA) (1:1500,14395-1-AP, Proteintech, Wuhan, China), CD3 (1:500, GB15064, Servicebio, Wuhan, China), and CD68 (1:200, GB113109, Servicebio, Wuhan, China), respectively, and finally with Alexa Fluor^®^ 488-conjugated goat anti-rabbit IgG (1:500, GB25303, Servicebio, Wuhan, China). Thereafter, nuclei were stained with 4, 6-diamino-2-phenylindole (DAPI, G1012, Servicebio, Wuhan, China).

Cellular immunofluorescence assay was performed using the same method described above. Briefly, 4% paraformaldehyde was used to fixed RAW264.7 cells at room temperature for 15 min. After incubation overnight at 4 °C with anti-iNOS antibody (1:500, GB11119, Servicebio, Wuhan, China) or anti-NF-κB p65 antibody (1:100, T55034, Abmart, Shanghai, China), slides of cells were incubated with Cy3-conjugated goat anti-rabbit IgG (1:500, GB21303, Servicebio, Wuhan, China) or Alexa Fluor^®^ 488-conjugated goat anti-rabbit IgG (1:500, GB25303, Servicebio, Wuhan, China) for 2 h at room temperature. Subsequently, nuclei were stained with DAPI (G1012, Servicebio, Wuhan, China). Both tissues and cells were examined under a fluorescence microscope, and their images were captured by a digital slide scanner (Pannoramic 250 FLASH, Budapest, Hungary). Two researchers who were blinded to the experimental grouping, assessed three random fields of views per slide. Quantitative data is calculated using the Image J software.

### Culture of RAW 264.7 macrophage cell line

The mouse macrophage cell line RAW264.7 was purchased from Wuhan Pricella Biotechnology Ltd (CL-0190, Servicebio, Wuhan, China). RAW264.7 cells were cultured in Dulbecco’s modified Eagle’s medium (DMEM, HyClone, Logan, USA) supplemented with 10% fetal bovine serum (FBS, Gibco, New York, USA) and 1% streptomycin/penicillin (Servicebio, Wuhan, China) in a humidified incubator with 5% CO_2_ at 37℃.

In in vitro experiment, IL-7 (HY-P73226, MedChemExpress, New Jersey, USA) was used to simulate a pathological environment. Cells in the IL-7 groups were treated with IL-7 (10 ng/mL) for 24 h as described in previous studies (Kim et al. 2017; Li et al. 2012). Cell experiments were divided into two parts. The first part included the following groups: (i) PBS + shRNA, (ii) IL-7 + shRNA, (iii) PBS + shIL-7R, and (iv) IL-7 + shIL-7R. The second part included the following groups: (i) PBS + shRNA + pCMV-NC, (ii) IL-7 + shRNA + pCMV-NC, (iii) IL-7 + shIL-7R + pCMV-NC, (iv) IL-7 + shRNA + pCMV-IL-7R, and (v) IL-7 + shRNA + pCMV-IL-7R + BAY 11–7082. BAY 11–7082 (a NF-κB inhibitor, HY-13453, MedChemExpress, New Jersey, USA) was added to the medium prior to stimulating the cells with IL-7.

### Plasmid transfection

The plasmids used to knock down (shIL-7R) or overexpress (pCMV-IL-7R) IL-7R in cells and the corresponding negative-control plasmids (shRNA and pCMV-NC, respectively) were purchased from MiaoLingBio (Wuhan, China). RAW264.7 cells were seeded in 6-well plates. Upon reaching 40–50% confluence, the cells were transfected with the plasmid using Lipofectamine 3000 (Thermo Fisher Scientific, Massachusetts, USA) according to the manufacturer’s instructions. After 24 h of culture, the medium was replaced with a fresh complete medium. The culture was continued for 48 h for further experiments.

### Cell viability assay

Cell viability was tested using Cell Counting Kit-8 (CCK-8, G4103, Servicebio, Wuhan, China). Briefly, RAW264.7 cells (100 *µ*L) were seeded in 96-well plates at a density of 1 × 10^5^ cells/mL. 10 *µ*L of CCK-8 reagent was added to each well, and the plate was incubated for 2 h. Finally, absorbance at 450 nm was measured by an enzyme marker.

### Transwell assay

Cell migration capacity was tested by Transwell assay. In short, 100 µL of a RAW264.7 cell suspension treated with different plasmids (1 × 10^5^ cells) was inoculated in an upper Transwell chamber (8-*µ*m wells, 24-well plate; Corning, New York, USA), followed by the addition of serum-free DMEM supplemented with PBS or IL-7. BAY 11–7082 was added to the culture medium before IL-7 stimulation based on the grouping. At the same time, 700 µL of DMEM supplemented with 10% FBS was added to the lower chamber. The cells remaining on the top surface of the polycarbonate insert were gently removed with a cotton swab after 24 h of incubation. The cells moved to the lower membrane were fixed with 4% paraformaldehyde for 15 min and stained with 0.1% crystal violet for 15 min at room temperature. Images were recorded with a light microscope. The migrated cell numbers were calculated in three random fields of view.

### Real-time reverse transcription polymerase chain reaction

Total RNA was extracted from mouse abdominal aortic tissues and RAW264.7 cells by Trizol reagent (Invitrogen, Massachusetts, USA) according to the manufacturer’s instructions. The integrity and purity of the extracted RNA were assessed using a spectrophotometer. The cDNA synthesis kits (G3333, Servicebio, Wuhan, China) were used to reverse transcribe a total of 1 *µ*g of extracted RNA. Subsequently, quantitative polymerase chain reaction (qPCR) was performed with SYBR Green qPCR Master Mix (G3328, Servicebio, Wuhan, China) and the Step-One™ Real-Time PCR system (Life Technologies, Massachusetts, USA). The relative gene expression level was calculated using the 2 − ΔΔCT method, with glyceraldehyde-3-phosphate dehydrogenase (GAPDH) serving as the internal control. The primer sequences (Servicebio, Wuhan, China) are as follows: IL-7 forward, 5’-CGTCGGGTTAGCTAGCATAGC-3’; IL-7 Reverse, 5’- TGCTGACGCCTAGCATCGATAC-3′; IL-7R forward, 5’-CCCTCGTGGAGGTAAAGTGC‐3’; IL-7R reverse, 5’-CCTTCCCGATAGACGACACTC-3’; iNOS forward, 5’-GTTCTCAGCCCAACAATACAAGA‐3’; iNOS reverse, 5’-GTGGACGGGTCGATGTCAC‐3’; TNF-α forward, 5’-CCCTCACACTCAGATCATCTTCT-3’; TNF-α reverse, 5’-GCTACGACGTGGGCTACAG-3’; IL-6 forward, 5’-TAGTCCTTCCTACCCCAATTTCC-3’; IL-6 reverse, 5’-TTGGTCCTTAGCCACTCCTTC-3’; CD206 forward, 5’‐CTCTGTTCAGCTATTGGACGC‐3’; CD206 reverse, 5’‐CGGAATTTCTGGGATTCAGCTTC‐3’; Fizz1 forward, 5’‐CCAATCCAGCTAACTATCCCTCC‐3’; Fizz1 reverse, 5’‐ACCCAGTAGCAGTCATCCCA‐3’; Arg-1 forward, 5’‐CAGAAGAATGGAAGAGTCAG‐3’; Arg-1 reverse, 5’‐CAGATATGCAGGGAGTCACC‐3’; GAPDH forward, 5’-TGTGGGCATCAATGGATTTGG-3’; GAPDH reverse, 5’-ACACCATGTATTCCGGGTCAAT-3’.

### Western blot analysis

Total proteins were extracted from mouse abdominal aortic tissues and RAW264.7 cells using RIPA lysis buffer at 4 °C. The BCA protein assay kits (P0010, Beyotime, Shanghai, China) were used to evaluated the concentration of the extracted proteins. Equal amounts of protein extracts were separated via sodium dodecyl sulfate-polyacrylamide electrophoresis (SDS-PAGE, 10% gels) and transferred to nitrocellulose (NC) membranes (Millipore, Massachusetts, USA). The NC membranes were blocked by 5% skimmed milk for 1 h at room temperature and then incubated with corresponding primary antibodies overnight at 4 °C. The following antibodies were used: anti-IL-7R (1:200, TD6362, Abmart, Shanghai, China ), anti-TNF-α (1:1000, Cat#YM3478, Immunoway, Texas, USA, ), anti-MMP9 (1:1000, 10375-2-AP. Proteintech, Wuhan, China), anti-Arg-1 (1:1000, 16001-1-AP, Proteintech, Wuhan, China), anti-IκB alpha (1:1000, T55026, Abmart, Shanghai, China), anti-Phospho-IκB alpha (1:1000, TP56280, Abmart, Shanghai, China), anti-NF-κB p65 (1:1000, T55034, Abmart, Shanghai, China), anti-Phospho-NF-κB p65 (Ser536, 1:1000, TP56372, Abmart, Shanghai, China) and anti-GAPDH (1:5000, 60004-1-Ig, Proteintech, Wuhan, China). The following day, the membrane was incubated with species-appropriate HRP-conjugated secondary antibodies (1:3000, GB23303, GB23301, Servicebio, China) for 1–2 h at room temperature. Immunoreactive bands were visualized by the enhanced chemiluminescence (ECL) reagent (BL520, Biosharp, Beijing, China) and proteins were quantified with the Image J software. The data were normalized to that of GAPDH.

### Statistical analysis of data

All data were obtained from at least three independent experiments and were expressed as the mean ± SEM. The Shapiro–Wilk test was used to assess the normality of continuous data. Comparison of data among multiple groups was performed using one-way analysis of variance (ANOVA) with Tukey’s multiple comparison test or Brown–Forsythe ANOVA. AAA incidence was compared using Fisher’s exact test. Survival rates were analyzed via Kaplan–Meier analysis and compared using the log-rank test. The Kruskal–Wallis test was used to compare the degree of elastin degradation. Two researchers who were blinded to the experimental grouping statistically analyzed the data using the GraphPad Prism software (version 10.0, San Diego, USA). A p-value of < 0.05 was considered statistically significant.

## Result

### IL-7R was upregulated in AAAs and influenced the development of AAAs in mice

**Fig. 1:**
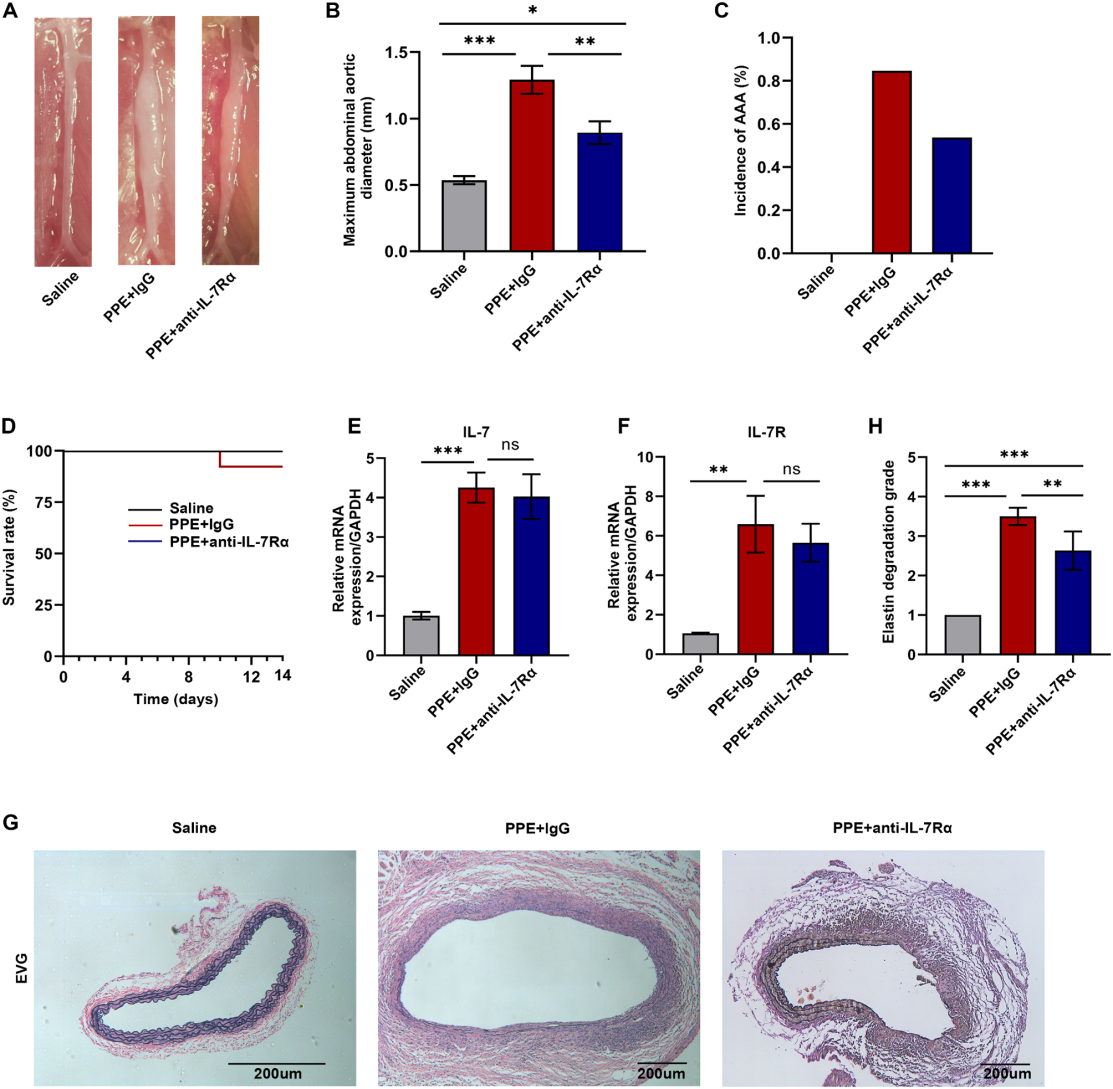
Inhibition of IL-7R can slow down the expansion of abdominal aorta in AAA mice. (**A**) Representative images of the subrenal abdominal aorta of WT mice treated with saline or PPE (Saline, *n* = 8; PPE + lgG, *n* = 13; PPE + anti-IL-7Rα, *n* = 13); (**B**) The maximum abdominal aortic diameter in each group of mice after 14 days of exposure to saline or PPE; (**C**) Incidence of AAA formation in each group; (**D**) Kaplan-Meier survival curve of mice in each group; (**E, F**) Relative mRNA expression of IL-7 and IL-7R in the abdominal aorta in each group (*n* = 3); (**G, H**) Representative images and statistical analysis of EVG staining for abdominal aortic tissue in control, PPE + lgG, and PPE + anti-IL-7Rα groups. Scale bar = 200 *µ*m. Data are expressed as mean ± SEM. *P < 0.05, **P < 0.005, ***P < 0.001

To examine the role of IL-7R in AAAs, we randomly divided C57BL/6 mice into three groups as follows: control, PPE + IgG, and PPE + Anti-IL-7Rα. PPE was used to establish mouse models of AAA, and the effects of IL-7R inhibition (anti-IL-7Rα mAb) on the development of AAAs were investigated in these models. Compared with the control group, the PPE + IgG group had a significantly larger diameter of the abdominal aorta. Although treatment with anti-IL-7Rα mAb significantly suppressed the expansion of the abdominal aorta in mice treated with PPE (0.89 ± 0.30 mm vs. 1.32 ± 0.38 mm, Fig. 1A and B), it did not affect the incidence of AAA or the survival rates of mice with AAAs. There were no significant differences in the incidence of AAA between the PPE + IgG and the PPE + anti-IL-7Rα groups (84.6% versus 53.8%, *P* = 0.364; Fig. 1C and D). qRT-PCR confirmed that the mRNA expression of IL-7R was significantly higher in mice with AAAs; however, treatment with anti-IL-7Rα mAb did not influence IL-7R expression in mice with AAAs (Fig. 1E and F). Furthermore, we used EVG staining to evaluate pathological changes and elastin degradation in mouse aortic tissues. The results showed that inhibition of IL-7R delayed the progression of AAAs (Fig. 1H and G). These data suggest that inhibition of IL-7R can inhibit the development of AAAs.

### IL-7R regulated the progression of AAAs by targeting macrophages

**Fig. 2:**
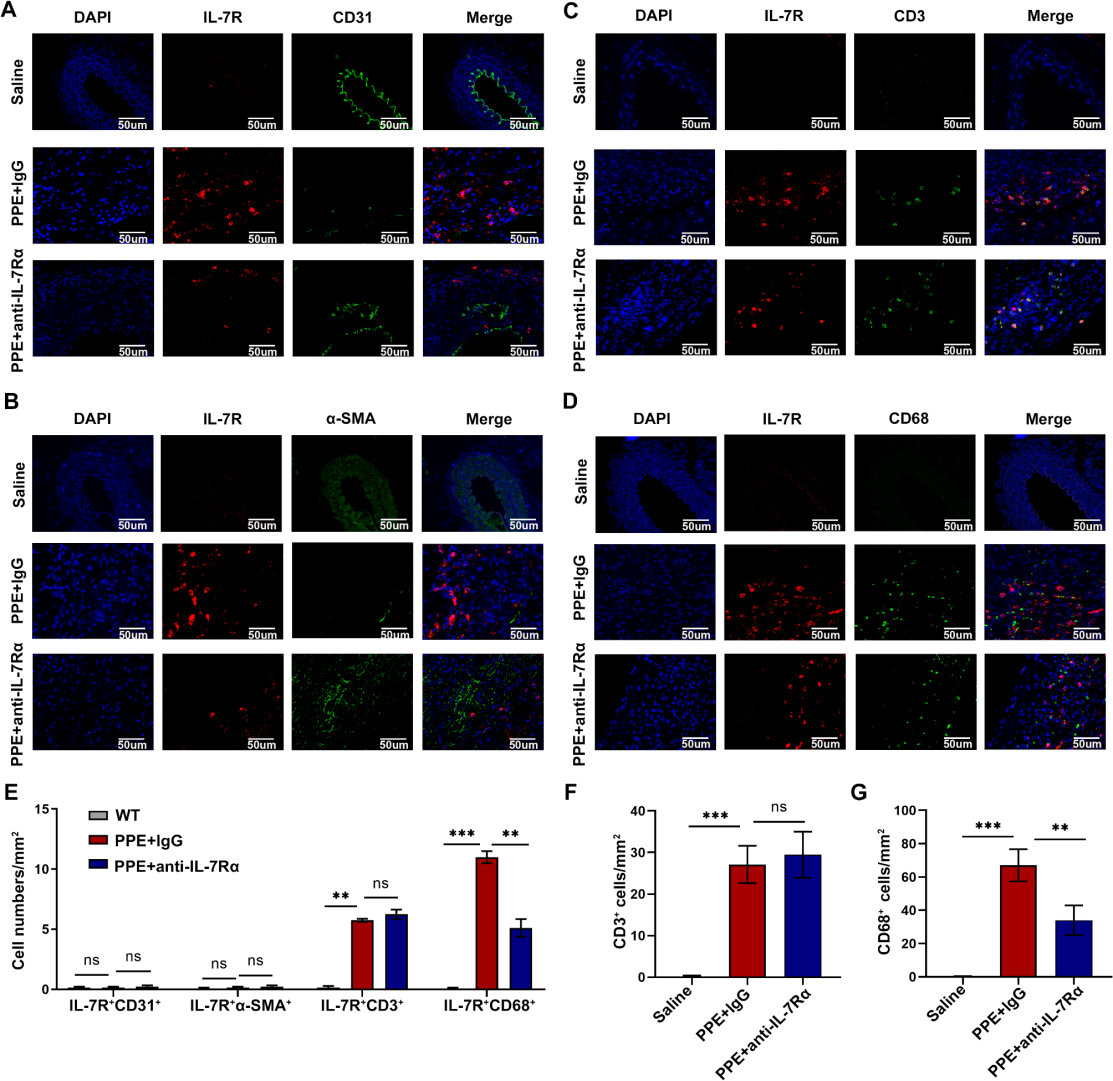
IL-7R regulatedz the progression of AAA by targeting macrophages. (**A-D**) Representative images of double immunofluorescence staining for CD31 (green), α-SMA (green), CD3 (green), CD68 (green) and IL-7R (red) in the abdominal aorta of mice in control, PPE + lgG, and PPE + anti-IL-7Rα groups, scale bar = 50 *µ*m. (**E**) Quantitative statistics of double immunofluorescence staining (*n* = 3). (**F-G**) Quantification of CD3^+^ T-cell (**F**) and CD68^+^ macrophage (**G**) numbers (*n* = 3). Data are expressed as mean ± SEM. *P < 0.05, **P < 0.005, ***P < 0.001

Four cell types, namely, endothelial cells (Franck et al. 2013; Mayr and Watkins et al. 2013; Liu et al. 2021), smooth muscle cells (Sun et al. 2022; Lu et al. 2020; Zhang et al. 2023), T lymphocytes (Li et al. 2022; Lu et al. 2022) and macrophages (Raffort et al. 2017; Davis et al. 2021; Filiberto et al. 2022) are mainly associated with the AAA development. To assess the relationship between IL-7R and these four cell types in mice with AAAs, we used double immunofluorescence staining to locate the expression of IL-7R in mouse abdominal aortic tissues and labelled these cells with CD31, α-SMA, CD3, and CD68, respectively (Fig. 2A-D). The results showed that IL-7R was expressed in lymphocytes and macrophages (Fig. 2E). Notably, treatment with anti-IL-7Rα mAb significantly decreased the proportion of infiltrating macrophages but did not affect the proportion of infiltrating T lymphocytes in the aortic tissues of mice with AAAs (Fig. 2F and G). Therefore, we hypothesized that IL-7R might function in the development of AAAs primarily through macrophages.

### Knockout of IL-7R inhibited inflammation and the development of AAAs

**Fig. 3:**
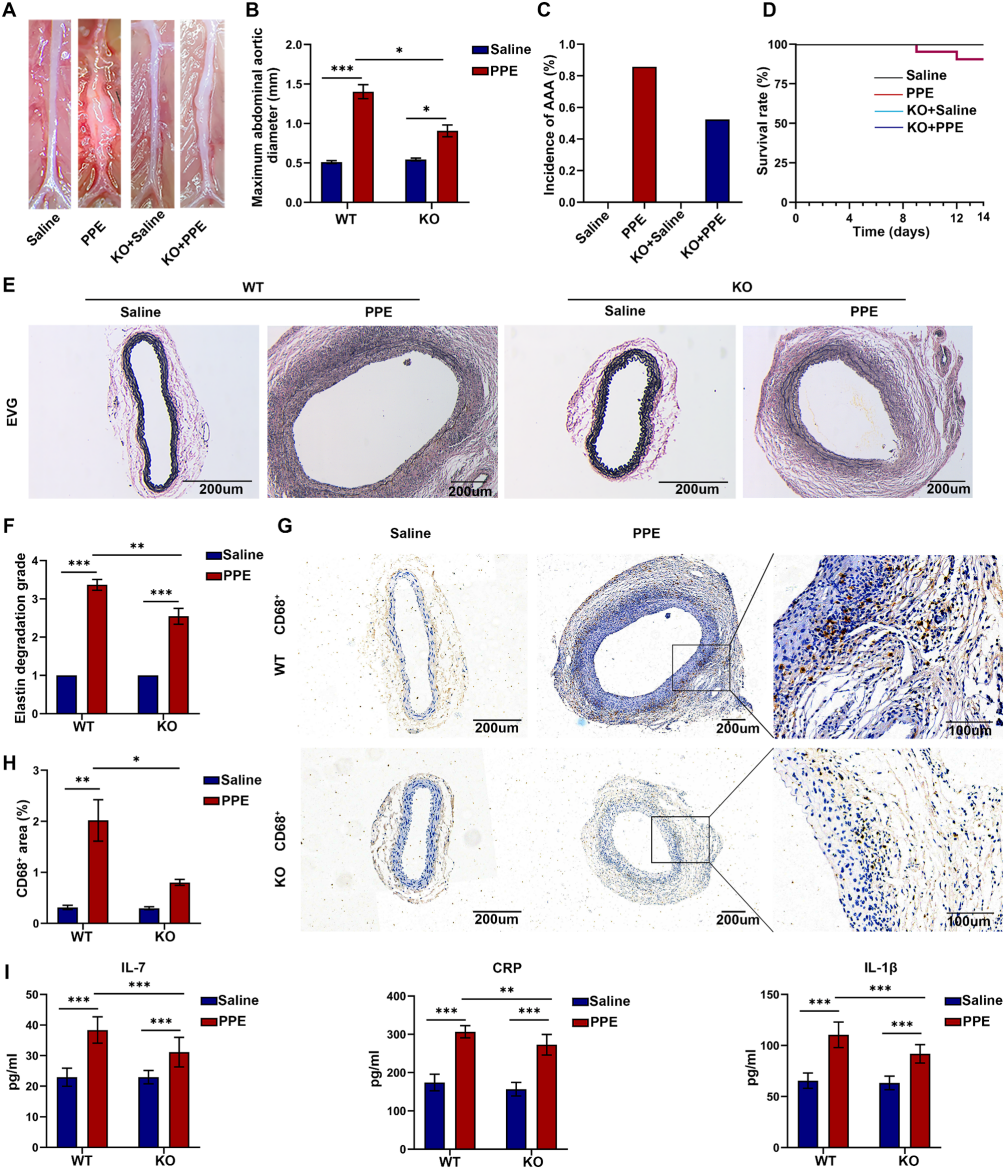
Knockout of IL-7R reduced macrophage infiltration and inhibited AAA development. (**A**) Representative images of the subrenal abdominal aorta in mice of Saline (*n* = 9), PPE (*n* = 21), KO + Saline (*n* = 9), KO + PPE (*n* = 21) group; (**B**) The maximum abdominal aortic diameter in each group; (**C**) The incidence of AAA formation in each group; (**D**) Kaplan-Meier survival curve of mice in each group; (**E-F**) EVG staining was used to evaluate the degree of elastin degradation. Representative images of EVG staining (**E**) and statistical analysis of elastin degradation (**F**) in abdominal aortic tissue of mice in each group (*n* = 3), scale bar = 200 *µ*m; (**G**) Representative immunohistochemical staining of macrophages (CD68^+^ cells) in abdominal aortic tissue of mice in each group. The illustration shows a high-power image of the macrophages. Scale bar = 200 *µ*m and 100 *µ*m; (**H**) Quantification of CD68^+^ positive area in aorta of each group (*n* = 3); (**I**) Serum levels of IL-7, CRP and IL-1β in each group were detected by ELISA (*n* = 6). Data are expressed as mean ± SEM. *P < 0.05, **P < 0.005, ***P < 0.001

To further explore the effects of IL-7R on macrophages in the development of AAAs, we used IL-7R-knockout mice and their WT littermates to establish AAA models (Fig.S1A). IL-7R knockout resulted in a decrease in the maximum diameter of the abdominal aorta in mice with AAAs (1.40 ± 0.40 mm vs. 0.90 ± 0.33 mm, Fig. 3A and B). However, the incidence of AAA (85.7% versus 52.4%, *P* = 0.193; Fig. 3C) and the survival rates (Fig. 3D) of mice with AAAs were not significantly different between the PPE + WT and PPE + KO groups. Subsequently, EVG staining of abdominal aortic tissues showed that the extent of elastin degradation was lower in the IL-7R KO group than in the WT group (Fig. 3E and F). Immunohistochemical staining showed increased macrophage infiltration in abdominal aortic tissues after 14 days of exposure with PPE, IL-7R knockout was beneficial in reducing this infiltration (Fig. 3G and H). ELISA showed that the serum levels of IL-7, CRP, and IL-1β were lower in the PPE + KO group than in the PPE + WT group, indicating an attenuated inflammatory response in the PPE + KO group (Fig. 3I).

### Knockout of IL-7R regulated macrophage polarization by inhibiting the NF-κB pathway

**Fig. 4:**
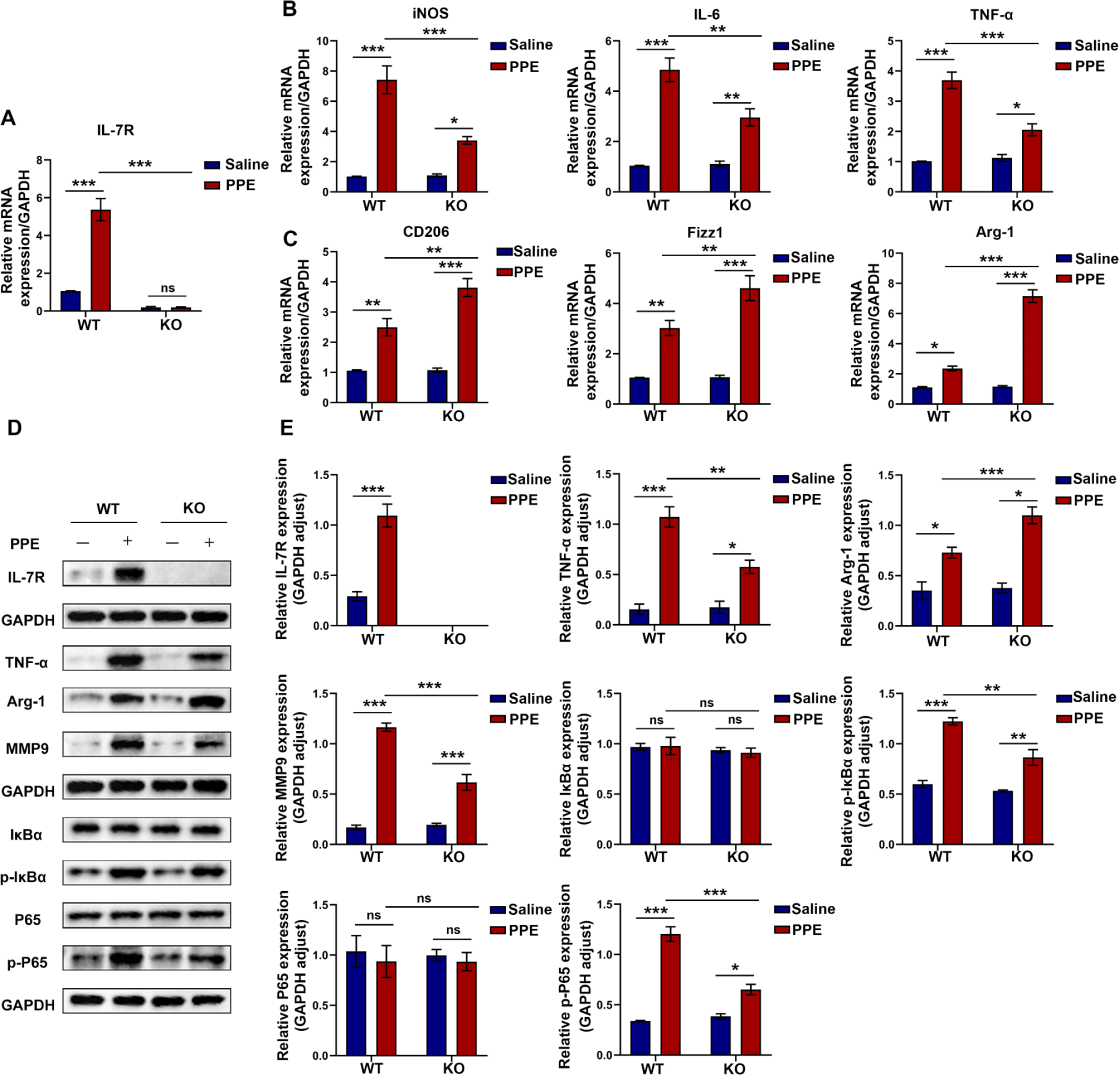
Knockout of IL-7R regulated polarization of macrophages by inhibiting the NF-κB pathway. (**A**) The relative mRNA expression of IL-7R in the abdominal aorta of mice in each group (*n* = 3); (**B**) The relative mRNA expression levels of iNOS, IL-6, and TNF-α in each group (*n* = 3); (**C**) The relative mRNA expression levels of CD206, Fizz1, and Arg-1 in each group (*n* = 3); (**D**) Representative western blotting images of IL-7R, TNF-α, Arg-1, MMP9, IκBα, p-IκBα, P65, and p-P65 in mouse aortic tissues from each group; (**E**) Protein expression of IL-7R, TNF-α, Arg-1, MMP9, IκBα, p-IκBα, P65, and p-P65 relative to GAPDH (*n* = 3). Data are expressed as mean ± SEM. *P < 0.05, **P < 0.005, ***P < 0.001

qRT-PCR showed that in PPE-treated mice, the mRNA expression of M1-related molecules iNOS, IL-6 and TNF-α was down-regulated in the KO group compared with those WT littermate mice, and the mRNA expression of M2-related molecules CD206, Arg-1 and Fizz1 was increased (Fig. 4A-C). Conclusively, the IL-7/IL-7R axis leads to M1-like macrophage polarization, exacerbating the expansion of AAAs. Subsequently, western blotting was used to analyze the protein expression of macrophage markers in the abdominal aortic tissues of mice with AAAs (Fig. 4D and E). Compared with the PPE + WT group, the PPE + KO group had significantly higher protein expression of Arg-1 but lower protein expression of TNF-α and MMP9. These results indicated a decrease in the infiltration of pro-inflammatory macrophages and an increase in the infiltration of anti-inflammatory macrophages in the PPE + KO group relative to the PPE + WT group, suggesting that IL-7R may influence the polarization of macrophages. Given that the NF-κB signaling pathway is important for the activation of M1 macrophages and the progression of AAAs (Kong et al. 2016; Cai et al. 2023), we analyzed the protein expression of NF-κB pathway-related factors in the aortas of mice in the different groups via western blotting. As shown in Fig. 4D and E, treatment with PPE significantly enhanced the phosphorylation of IκB and NF-κB. However, knockout of IL-7R counteracted this change, indicating that IL-7R may regulate the activation of the NF-κB signaling pathway. Altogether, deficiency of IL-7R in mice alleviated the development of AAAs, influenced the polarization of macrophages, reduced the infiltration of macrophage, and inhibited the activation of the NF-κB pathway.

### Knockdown of IL-7R inhibited macrophage infiltration, polarization, and migration by inhibiting the NF-κB pathway in vitro

**Fig. 5:**
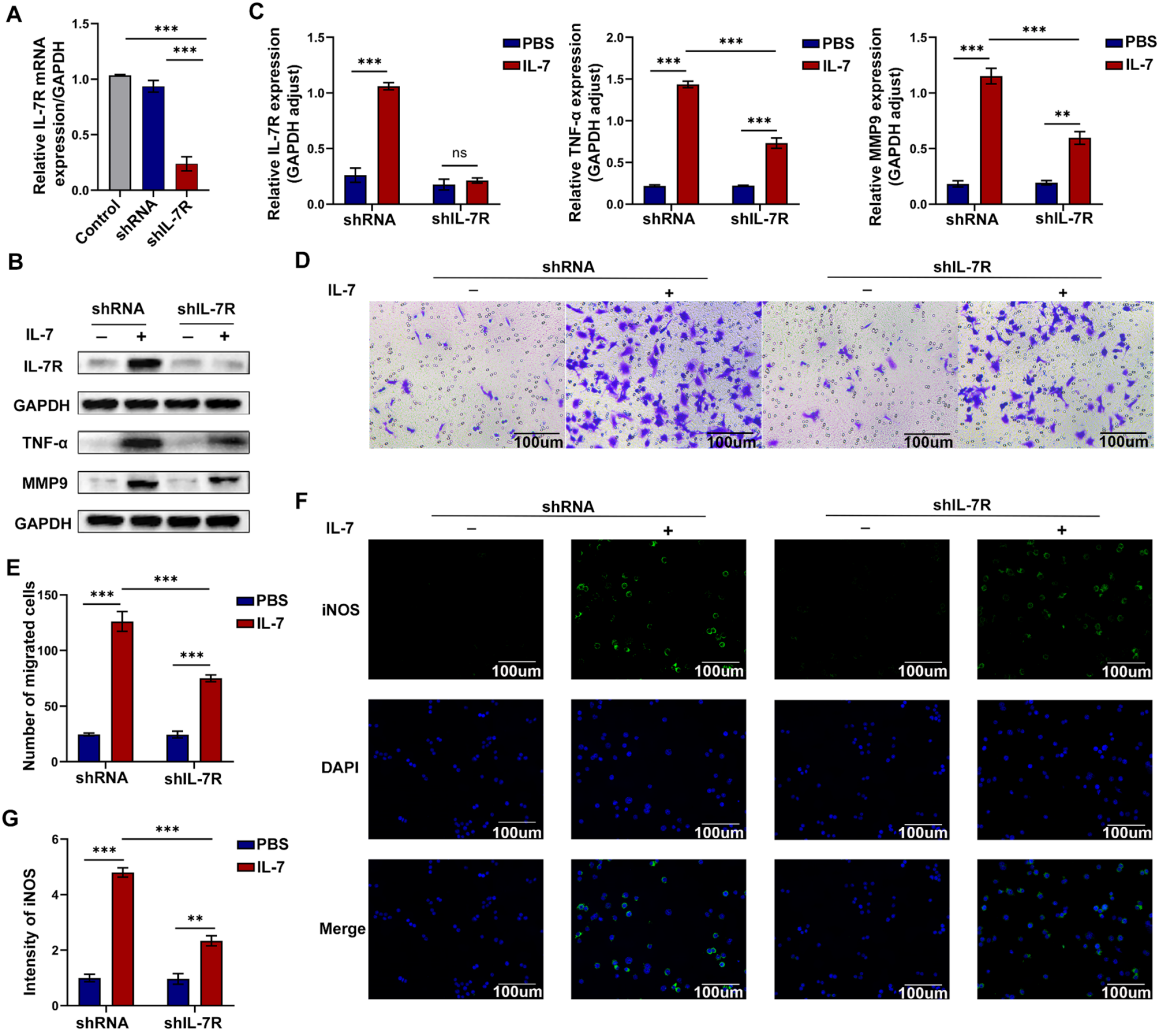
Deficiency of IL-7R inhibited the infiltration, polarization and migration of macrophages in vitro. (**A**) The relative mRNA expression level of IL-7R normalized to GAPDH in RAW264.7 cells transfected with shIL-7R or shRNA (*n* = 3); (**B**) Western blotting images for protein expression of IL-7R, TNF-α, and MMP9 in each cell group; (**C**) Expression of IL-7R, TNF-α and MMP9 relative to GAPDH (*n* = 3); (**D**) Transwell assay was performed to assess the migration of RAW264.7 cells in each group, scale bar = 100 *µ*m; (**E**) The number of migrated cells in each group was quantified by transwell assays (*n* = 3); (**F**) Representative images of iNOS (green) immunofluorescence staining. Nuclei were stained with DAPI (blue), scale bar = 100 *µ*m; (**G**) Statistical analysis diagram of iNOS immunofluorescence staining (*n* = 3). Data are expressed as mean ± SEM. *P < 0.05, **P < 0.005, ***P < 0.001

**Fig. 6:**
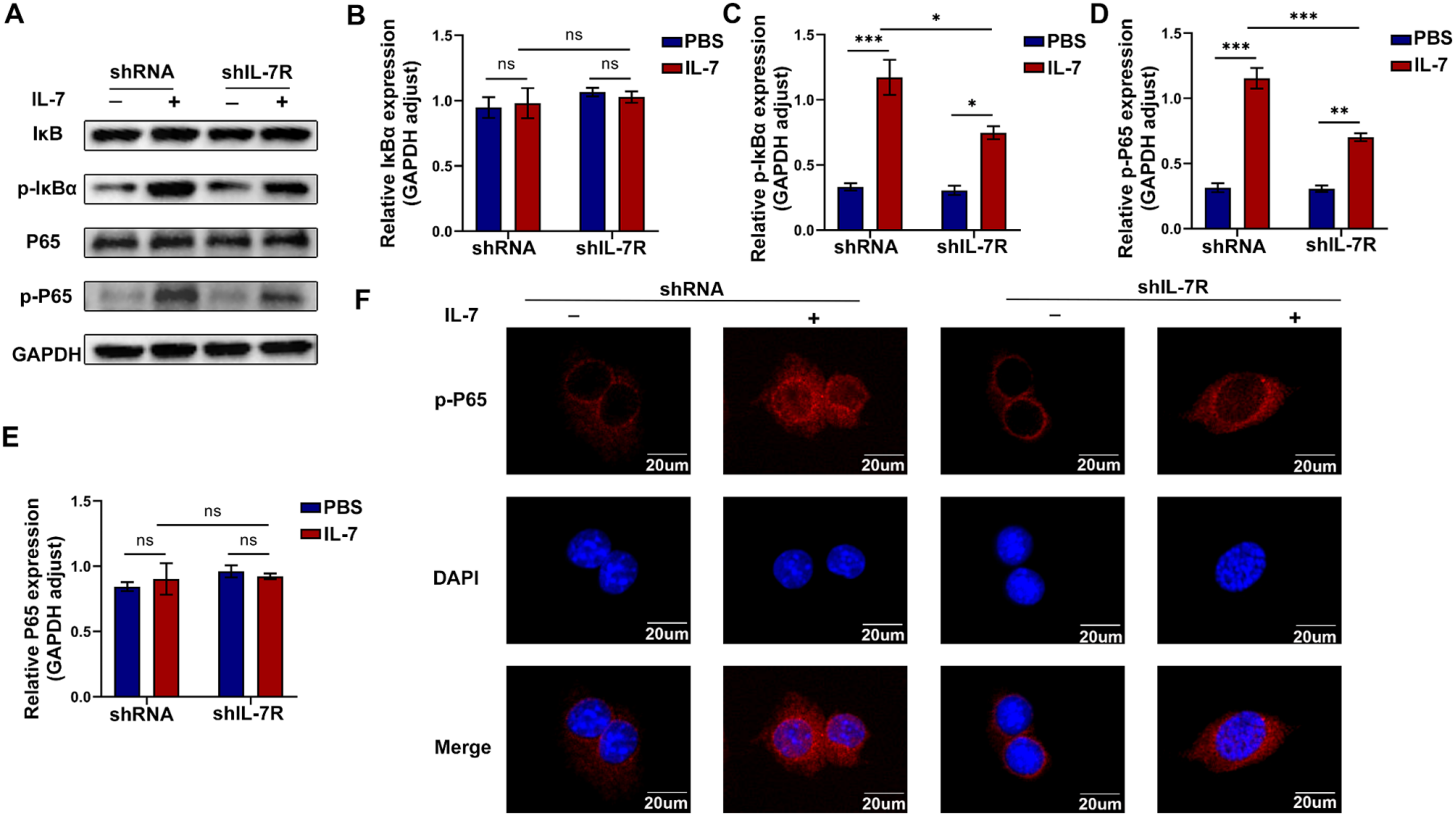
Deficiency of IL-7R suppressed the NF-κB pathway activation of macrophages in vitro. (**A**) Western blotting images for protein expression of IκBα, p-IκBα, P65 and p-P65 in each cell group; (**B-E**) Statistical analysis of IκBα (**B**), p-IκBα (**C**), P65 (**D**), p-P65 (**E**) protein expression levels relative to GAPDH (*n* = 3); (**F**) Representative images of p-P65 (red) immunofluorescence staining. Nuclei were stained with DAPI (blue), scale bar = 20 *µ*m. Data are expressed as mean ± SEM. *P < 0.05, **P < 0.005, ***P < 0.001

Based on the abovementioned results, we transfected RAW264.7 macrophages with shIL-7R or shRNA to verify the effects of IL-7R on macrophages in vitro. The viability of cells was detected by CCK-8 assay. The result showed that IL-7 treatment did not affect the viability of RAW264.7 cells (Fig.S1B). Compared with the blank control and shRNA groups, the shIL-7R group had significantly lower mRNA expression of IL-7R (Fig. 5A). To examine the relationship between IL-7R activation and macrophage polarization, we treated RAW264.7 cells with IL-7 to induce their polarization toward the M1 phenotype. The expression of IL-7R increased in response to IL-7 stimulation but decreased after transfection with shIL-7R (Fig. 5B and C). Western blotting showed that knockdown of IL-7R reversed the IL-7-induced increase in the protein expression of TNF-α and MMP9 in RAW264.7 cells (Fig. 5B and C). Furthermore, Transwell assay and immunofluorescence staining were performed to investigate the migration and polarization of RAW264.7 cells, respectively (Fig. 5D-G). The results displayed that the migratory ability of the shIL-7R group was weaker than that of the shRNA group after stimulation with IL-7. Moreover, knockdown of IL-7R resulted in a notable reduction in the expression of iNOS, a marker of M1 macrophages ((Fig. 5F and G). We assessed the relationship between IL-7R and the NF-κB pathway in RAW264.7 cells via western blotting and immunofluorescence staining. Western blotting showed that knockdown of IL-7R suppressed the phosphorylation of IκB and NF-κB (Fig. 6A-E). In the IL-7 + shIL-7R group, the positive p-P65 overlapping with DAPI was significantly decreased, indicating that more P65 was located in the cytoplasm in an inactive state and the activation of the NF-κB pathway was inhibited (Fig. 6F). These results suggested that deficiency of IL-7R inhibited the infiltration, polarization, and migration of pro-inflammatory macrophages through inhibition of the NF-κB pathway in vitro.

### Inhibition of the NF-κB signaling pathway blocked the effects of upregulated IL-7R activation on macrophages

**Fig. 7:**
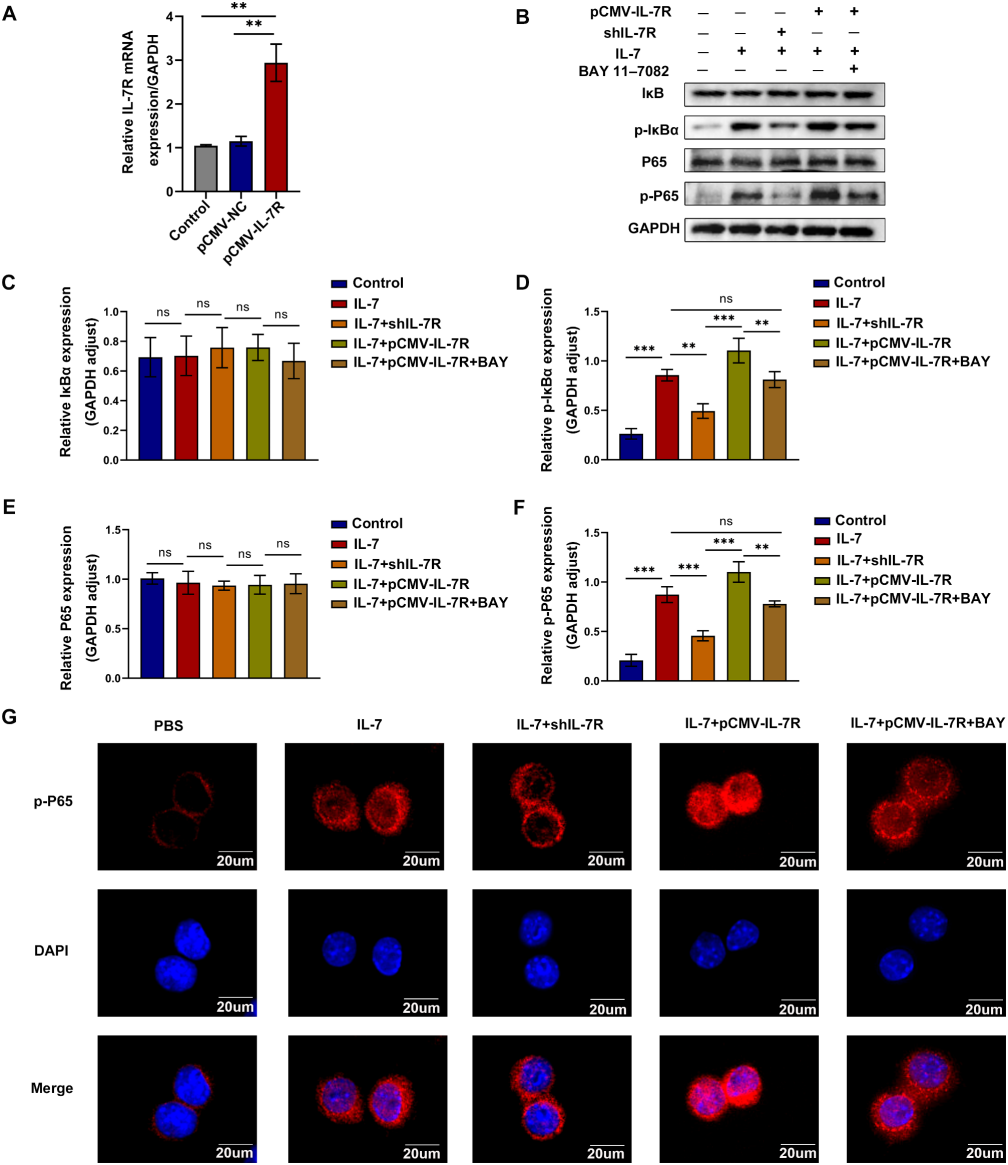
Overexpression of IL-7R upregulated the activation of NF-κB signaling pathway. (**A**) The relative mRNA expression level of IL-7R normalized to GAPDH in RAW264.7 cells transfected with pCMV-NC or pCMV-IL-7R (*n* = 3); (**B**) Western blotting images for protein expression of IκBα, p-IκBα, P65 and p-P65 in each cell group; **C-F**) Statistical analysis of IκBα (**C**), p-IκBα (**D**), P65 (**E**), p-P65 (**F**) protein expression levels relative to GAPDH (*n* = 3); (**G**) Representative images of p-P65 (red) immunofluorescence staining. Nuclei were stained with DAPI (blue), scale bar = 20 *µ*m. Data are expressed as mean ± SEM. *P < 0.05, **P < 0.005, ***P < 0.001

**Fig. 8:**
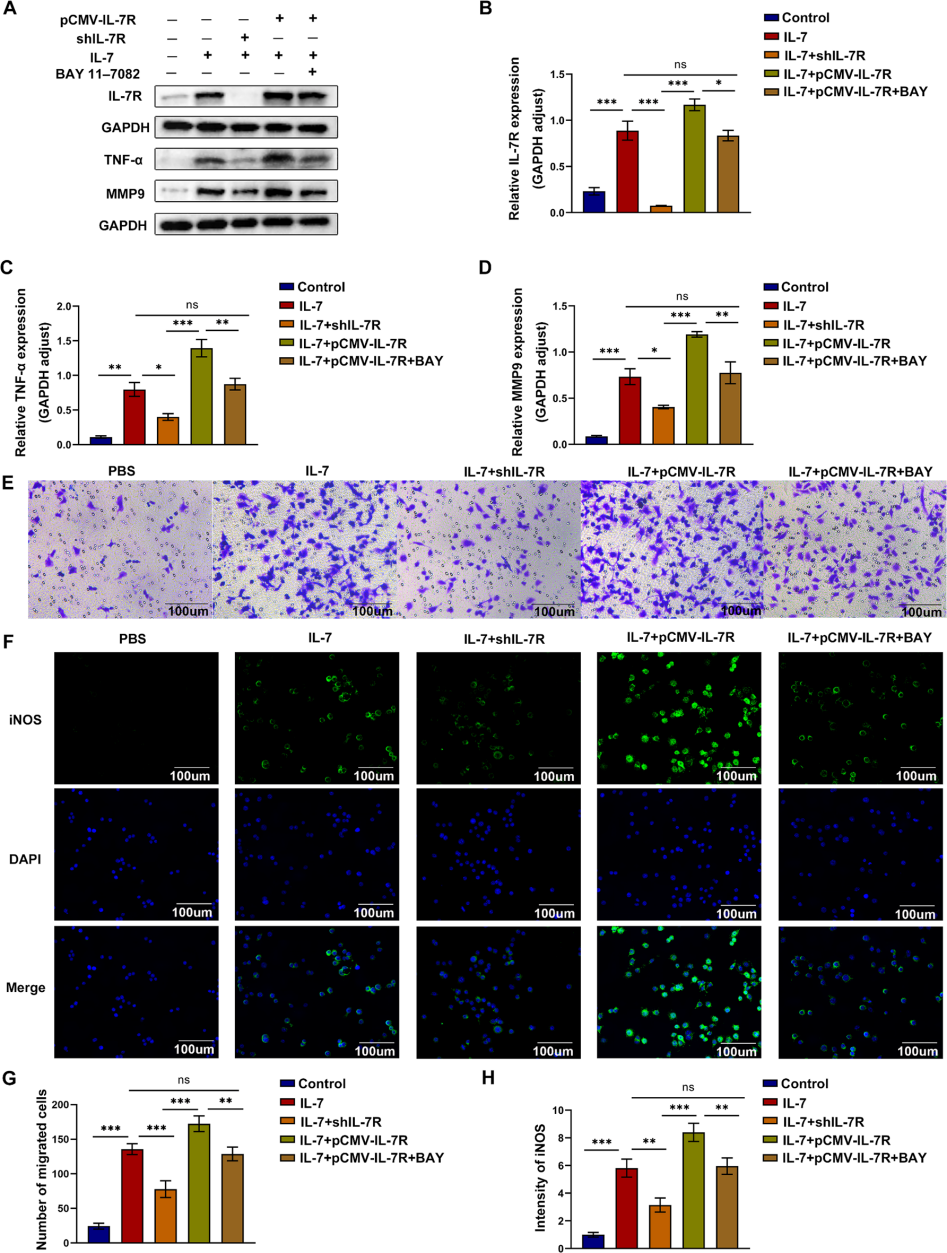
Inhibition of NF-κB signaling pathway blocked IL-7R overexpressed-induced macrophage polarization and migration in vitro. (**A**) Western blotting images for protein expression of IL-7R, TNF-α, MMP9 in each cell group; (**B-D**) Protein expression of IL-7R (**B**), TNF-α (**C**), and MMP9 (**D**) relative to GAPDH (*n* = 3); (**E**) Transwell assay assessed the migration of RAW264.7 cells in each group, scale bar = 100 *µ*m; (**F**) Representative images of iNOS (green) immunofluorescence staining. Nuclei were stained with DAPI (blue), scale bar = 100 *µ*m; (**G**) The number of migrated cells in each group was quantified by Transwell assays (*n* = 3); (**H**) Statistical analysis diagram of iNOS immunofluorescence staining (*n* = 3). Data are expressed as mean ± SEM. *P < 0.05, **P < 0.005, ***P < 0.001

To assess whether activated IL-7R promotes the infiltration, polarization, and migration of macrophages in an NF-κB-dependent manner, we transfected pCMV-IL-7R plasmids into RAW264.7 cells to overexpress IL-7R and inhibited the NF-κB pathway with BAY 11-7082. BAY 11-7082 inhibits the NF-κB signaling pathway by selectively and irreversibly inhibiting the phosphorylation of IκB. The viability of cells was detected by CCK-8 assay (Fig.S1C). The mRNA expression of IL-7R was higher in the pCMV-IL-7R group (Fig. 7A). When RAW264.7 cells were stimulated with IL-7, western blotting and immunofluorescence staining for detecting p-P65 validated that treatment with BAY 11-7082 partially blocked the effects of pCMV-IL-7R on RAW264.7 cells stimulated with IL-7 (Fig. 7B-F). Furthermore, M1 polarization and macrophages migration occurred more severely in the pCMV-IL-7R group; however, treatment with BAY 11-7082 counteracted these effects (Fig. 8A-E and G). Similarly, the expression of iNOS was notably upregulated in the IL-7 + pCMV-IL-7R group than in the IL-7 + pCMV-IL-7R + BAY 11-7082 group (Fig. 8F and H). Altogether, these findings suggest that IL-7R promotes macrophage infiltration, polarization, and migration by activating the NF-κB pathway.

## Discussion

This study showed that IL-7R expression was significantly high in the aortic tissues of mice with AAAs. Inhibition of IL-7R regulated the accumulation of macrophages at the lesion sites in mice with AAAs. Moreover, knockout of IL-7R delayed the progression of AAA and inhibited the NF-κB activation and the infiltration, polarization, and migration of macrophages.

AAAs have an extremely high mortality rate owing to abdominal aortic dissection (Baman and Eskandari 2022; Schanzer and Oderich et al. 2021; Golledge et al. 2019). Inflammatory cell infiltration and elevated levels of pro-inflammatory cytokines in the aorta play a crucial role in the development of AAAs in human and animal models (Golledge et al. 2023; Márquez-Sánchez and Koltsova 2022; Zhang et al. 2021; Liu et al. 2019). Increased expression of cytokines and cell adhesion markers contributes to the recruitment of immune cells to the vessel wall, which aggravates inflammation (Davis et al. 2019; Shimizu et al. 2006). In this study, inflammatory factors such as IL-1β and CRP were significantly upregulated in the sera of mice with PPE-induced AAAs. Increasing evidence suggests that regulating the inflammation-related gene expressions is a promising strategy for controlling the progression of AAAs (Wang et al. 2010; Johnston et al. 2013).

IL-7, a member of the IL-2 family, is considered to affect aberrant immune activity in autoimmune diseases, such as diabetes and multiple sclerosis (Harrison et al. 2012; Harley et al. 2007), and chronic inflammatory diseases, such as rheumatoid arthritis (Hartgring et al. 2006), ankylosing spondylitis (Gracey et al. 2016), and inflammatory bowel disease (Anderson et al. 2011; Barata et al. 2019), by binding to IL-7R. IL-7R consists of a common gamma chain (γc, CD132, IL2RG) of the cytokine receptor and a specific IL-7R alpha chain (IL-7Rα, CD127). Individuals with inflammatory bowel disease have high levels of IL-7 and IL-7R and high activity of the colon-specific mucosal IL-7R signaling pathway. IL-7Rα antagonists have been shown to inhibit innate and adaptive inflammatory responses in different experimental models of colitis (Belarif et al. 2019). Consistently, in this experiment, the expression of IL-7 in serum and IL-7R in abdominal aortic tissues was significantly upregulated in mice with AAAs. Inhibition and knockout of IL-7Rα notably inhibited the formation and development of PPE-induced AAAs in vivo. Previous studies on IL-7 have primarily focused on its effects on T and B lymphocytes. IL-7 is an important cytokine for T cell development and plays an important regulatory role in T cell homeostasis (Bikker et al. 2012). Recent studies have shown that the IL-7/IL-7R axis is critical in the activation of monocytes and macrophages and that activating IL-7R can enhance the cytotoxicity of macrophages (Kim et al. 2020; Zhang et al. 2022).

Macrophages are characterized by high plasticity and can be stimulated to polarize to different phenotypes under pathological conditions, primarily from the M0 phenotype to either the M1 or M2 phenotype. These phenotypic changes in macrophages contribute to different functions in inflammatory pathways and are important for maintaining immune balance (Diao et al. 2024; Han et al. 2019). To the best of our knowledge, we first demonstrated that IL-7R is increased in macrophages infiltrating the abdominal aortic tissues of mice with AAAs and primarily promotes the development of AAAs through macrophages. The decreased expression of iNOS, TNF-α, and MMP9 and the increased expression of Arg-1 in the aortic tissues of mice with AAA suggested that deficiency of IL-7R inhibited the activation of M1 macrophages. Moreover, knockdown of IL-7R in RAW264.7 macrophages suppressed the IL-7-induced polarization toward the M1 phenotype and reduced the migratory ability of macrophages in vitro. However, it is possible that the increase in M2 activation in AAA tissues resulted from improved pathology.

The NF-κB signaling pathway is a classical inflammatory pathway (Saito et al. 2013). In the absence of inflammatory stimuli, NF-κB is maintained in an inactive state in the cytoplasm through its binding to the inhibitor subunit IκB. In response to an inflammatory stimulus, proteolytic hydrolysis of IκB exposes the nuclear recognition site of NF-κB, which is stimulated to enter the nucleus, where it drives the transcription of many cytokine genes (Baldwin et al. 1996). Previous studies have shown that the NF-κB signaling pathway stimulates M1 polarization (Wang, Lu et al. 2022). Low shear stress can promote the activation and infiltration of pro-inflammatory macrophages, facilitating the formation of AAAs (Wei et al. 2024). Inhibition of NF-κB signaling prevents the activation of M1-type macrophages in thoracic aortic dissection (Han et al. 2024). The IL-7/IL-7R axis has been shown to activate NF-κB in prostate cancer cells. However, the role of activated IL-7R in the NF-κB pathway and macrophages is still unclear. Our study confirmed that IL-7R regulate macrophage polarization by activating the NF-κB signaling pathway in RAW264.7 cells. Deficiency of IL-7R suppressed the development of AAAs, influenced the polarization and infiltration of macrophages, and inhibited the activation of the NF-κB pathway. Moreover, inhibition of the NF-κB signaling pathway by BAY 11-7082 attenuated the macrophage-mediated inflammatory responses caused by IL-7R overexpression.

In conclusion, this study demonstrates that IL-7R deficiency attenuates the initiation and progression of AAAs. The IL-7/IL-7R axis promotes the polarization and migration of macrophage by activating the NF-κB signaling pathway, which may be associated with the development of AAAs. These findings provide a valuable theoretical basis and novel therapeutic targets for exploring drugs targeting AAAs.

## Limitations

This study has some limitations. AAA progress in vivo is associated with numerous cellular and humoral factors. Although cellular experiments showed that IL-7R deletion inhibits M1 macrophage activation through inhibition of the NF-κB signaling pathway, it remains to be demonstrated by additional in vivo research whether the effect of IL-7R on AAA progression in experimental animals is closely related to the macrophages and NF-κB pathway. Besides, the increase in M2 activation may be the result of improved AAA pathology rather than the same causation relationship. Studies have shown that inhibition of IL-7R may impair lymphocyte homeostasis and influence normal immune function. Therefore, further studies in macrophage-specific IL-7R deletion mouse models are needed to elucidate the role of IL-7R on macrophages in AAA and to evaluate the safety and efficacy of drugs targeting IL-7R. The lack of validation for the role of IL-7R in human AAAs is also a limitation of our study. These limitations offer areas for the future refinement and investigation of the pathological mechanisms of AAAs.

## Electronic supplementary material

Below is the link to the electronic supplementary material.

Supplementary Material 1

## Data Availability

No datasets were generated or analysed during the current study.

## Abbreviations

AAA: Abdominal aortic aneurysm
VSMC: Vascular smooth muscle cell
ECM: Extracellular matrix
MMP: Matrix metalloproteinase
M1 macrophages: Classically activated macrophages
M2 macrophages: Alternatively activated macrophages
IL-1β: Interleukin-1β
iNOS: Inducible nitric oxide synthase
IL-7: Interleukin-7
IL-7R: IL-7 receptor
NF-κB: The nuclear factor kappa-B
MCP-1: Monocyte chemoattractant protein-1
TNF-α: Tumor necrosis factor-alpha
IL-7R KO: IL-7R knockout
WT: Wild-type
PPE: Elastase
BAPN: β-aminopropionitrile
EVG: Elastin Van Gieson
ELISA: Enzyme-linked immunosorbent assay
CRP: c-reactive protein
DAB: 3,3’-diaminobenzidine
α-SMA: α-smooth muscle actin
DAPI: 4, 6-diamino-2-phenylindole
DMEM: Dulbecco’s modified Eagle’s medium
FBS: Fetal bovine serum
CCK-8: Cell Counting Kit-8
qPCR: quantitative polymerase chain reaction
GAPDH: glyceraldehyde-3-phosphate dehydrogenase
SDS-PAGE: Sodium dodecyl sulfate-polyacrylamide electrophoresis
ECL: Chemiluminescence
