# Supplementary material for: Deficiency of IL-7R attenuates abdominal aortic aneurysms in mice by inhibiting macrophage polarization towards M1 phenotype through the NF-κB pathway
Source: Mol Med. 2025 Apr 16;31:138. doi: 10.1186/s10020-025-01209-2 (PMC12004661; doi:10.1186/s10020-025-01209-2)
Supplement: Supplementary file 1 — Supplementary Material 1 [file 10020_2025_1209_MOESM1_ESM.docx]

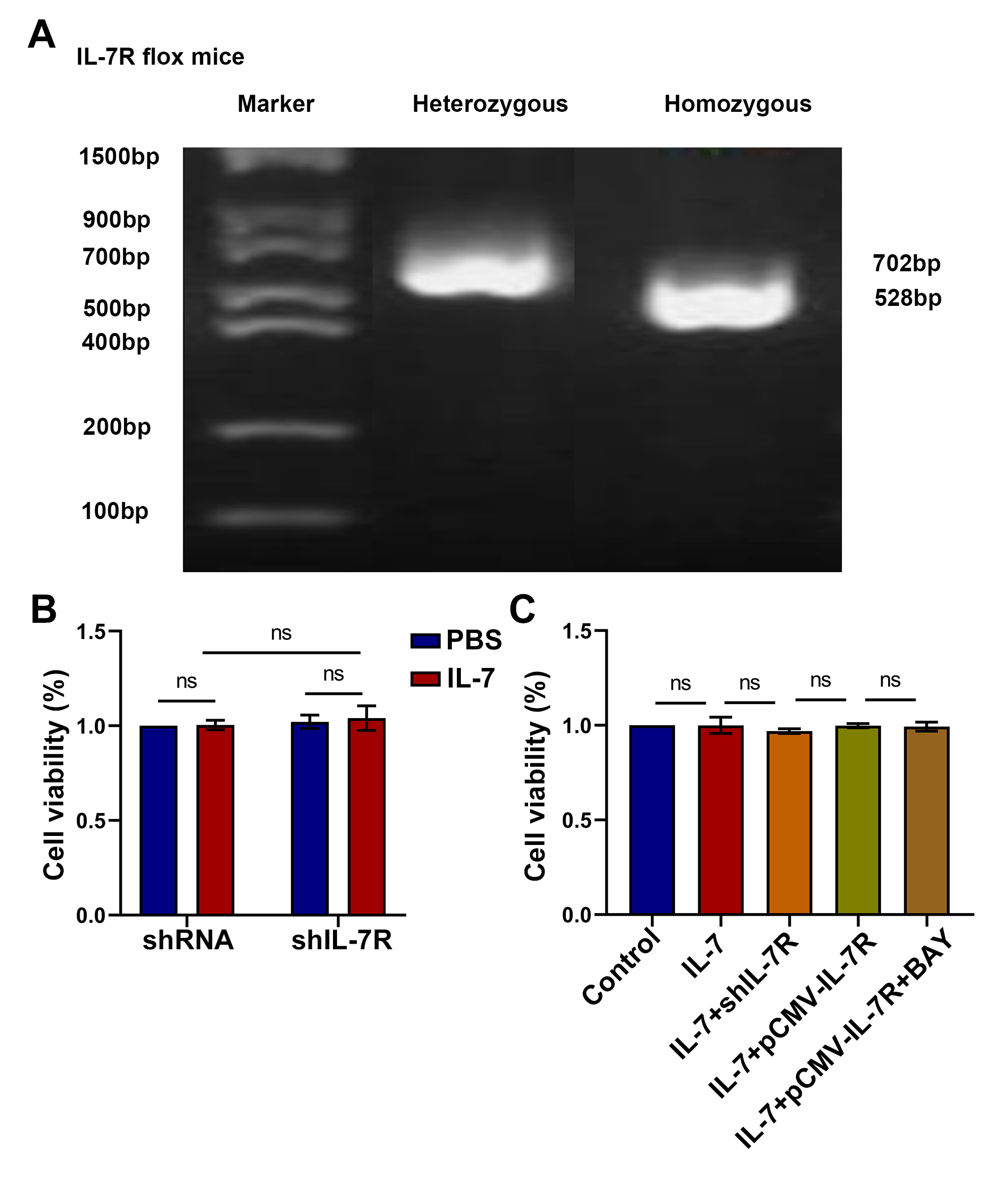


**Figure S1.** (A) Genotyping of IL-7R knockout mice. Detection of wild-type (WT) and IL-7R ^flox/flox^ strands. The expected PCR results are as follows: IL-7R ^flox/flox^ (homozygous) =702 bp, WT = 528 bp; (B) The viability of RAW264.7 cells was detected by CCK-8 assay; (C) The viability of RAW264.7 cells was detected by CCK-8 assay.


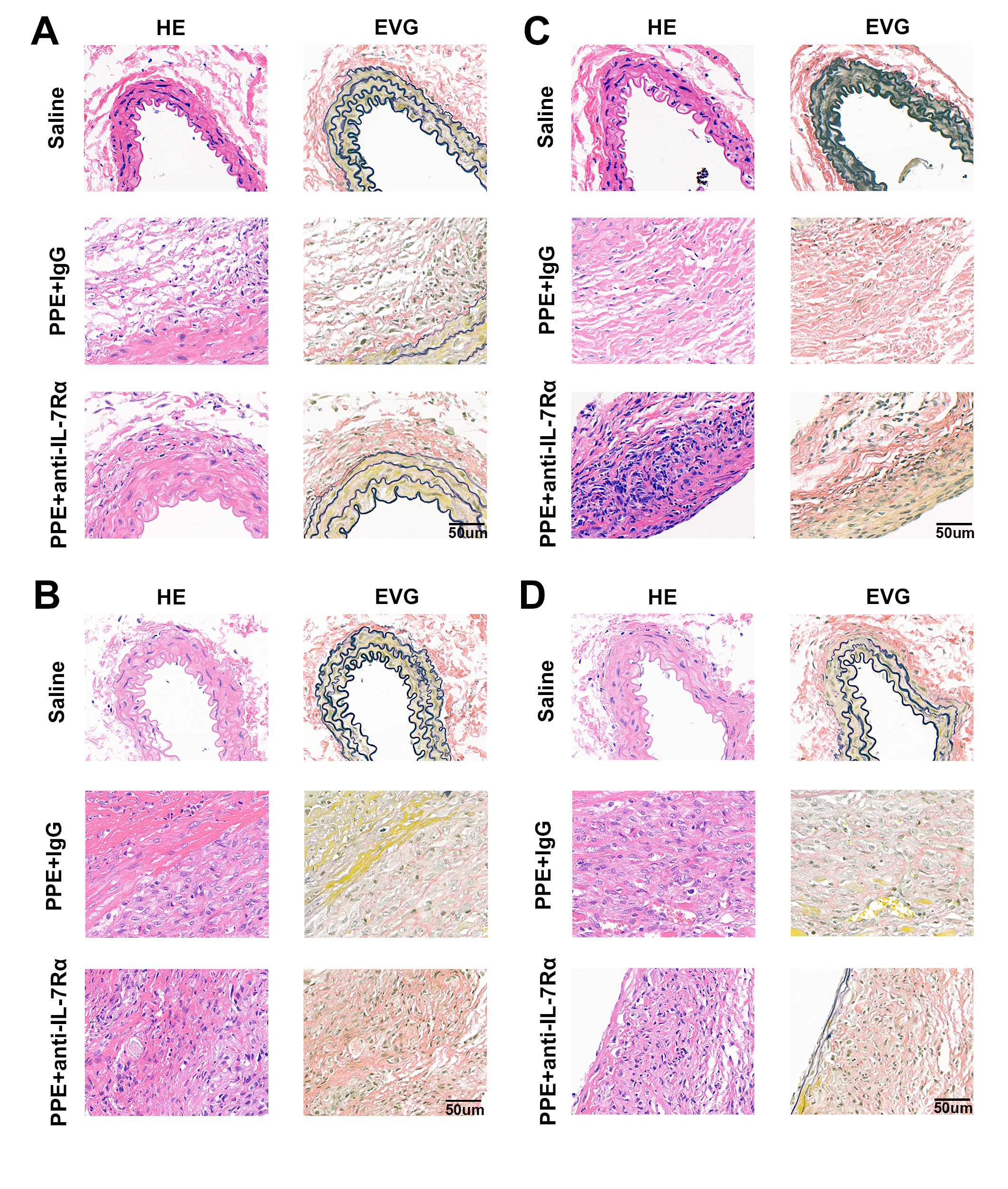


**Figure S2.** (A-D) Bright-field images of the same regions with HE and EVG staining of double immunofluorescence CD31 (A), α-SMA (B), CD3 (C), and CD68 (D) in Figure 2, , scale bar = 50 *μ*m.
